# Contrast-enhanced ultrasound-assisted catheter drainage for paravertebral tuberculous abscesses: a retrospective study of procedural safety

**DOI:** 10.3389/fmed.2026.1970327

**Published:** 2026-10-20

**Authors:** Dan Li, Peijun Chen, Wenzhi Zhang

**Affiliations:** Department of Ultrasonography, Hangzhou Red Cross Hospital (Integrated Chinese and Western Hospital of Zhejiang Province), Hangzhou, Zhejiang, China

**Keywords:** catheter drainage, contrast-enhanced ultrasound, paravertebral tuberculous abscess, spinal tuberculosis, ultrasound guidance

## Abstract

**Background:**

Paravertebral tuberculous abscess is a clinically important complication of spinal tuberculosis and may require image-guided drainage before definitive surgical management. Contrast-enhanced ultrasound (CEUS) may improve visualization of abscess vascularity and internal architecture, but its clinical value in catheter drainage for paravertebral tuberculous abscess remains insufficiently defined.

**Methods:**

This retrospective historical-control study included 67 patients with lumbar spinal tuberculosis complicated by paravertebral tuberculous abscess who underwent ultrasound-guided catheter drainage at Hangzhou Red Cross Hospital between January 2010 and January 2025. Patients were divided into a conventional ultrasound-guided drainage group (Group A, *n* = 39) and a CEUS-assisted drainage group (Group B, *n* = 28). Baseline characteristics, postoperative recovery parameters, inflammatory markers, pain scores, clinical outcomes, and drainage-related complications were compared between groups.

**Results:**

Postprocedural bleeding occurred after 5 of 41 drainage procedures (12.20%) in the conventional ultrasound-guided group and after 0 of 31 procedures (0.00%) in the CEUS-assisted group. Catheter occlusion and pericatheter purulent discharge rates did not differ between groups. Operative time, length of hospital stay, intraoperative blood loss, inflammatory markers, pain scores, cure rate, and recurrence rate also did not differ between groups.

**Conclusions:**

In this retrospective historical cohort, CEUS-assisted drainage was associated with fewer recorded postprocedural bleeding events and provided additional anatomical information for procedural planning.

## Introduction

According to the World Health Organization Global Tuberculosis Report 2025, China accounted for approximately 6.5% of newly reported tuberculosis cases worldwide in 2024 and remains a high-burden country (1). Delayed or advanced tuberculosis contributes to disability and continued transmission (2). Spinal tuberculosis is the most common form of osteoarticular tuberculosis and accounts for approximately 50% of musculoskeletal tuberculosis (3). Approximately 70%–80% of spinal tuberculosis extends along muscle planes or neurovascular pathways and forms a paravertebral tuberculous abscess (PTA) (4, 5). An enlarging PTA can cause pain, a palpable mass, spinal cord compression, and neurological deficits (6). Preoperative drainage can reduce abscess burden and relieve compression before definitive surgery. Ultrasound-guided catheter drainage is minimally invasive, repeatable, and provides real-time targeting. This study compared conventional ultrasound-guided drainage with contrast-enhanced ultrasound (CEUS)-assisted drainage in patients with PTA.

## Patients and methods

### Study design and patients

Patients who underwent ultrasound-guided catheter drainage for lumbar tuberculosis with PTA at Hangzhou Red Cross Hospital between January 2010 and January 2025 were eligible. Final inclusion required a positive Mycobacterium tuberculosis culture or Xpert result. We reviewed the electronic medical records, imaging studies, operative records, and follow-up records of 67 eligible patients. Data were extracted from February to April 2026 and analyzed from May to June 2026. No *a priori* sample size calculation was performed; all eligible patients treated during the study period were included.

Patients were grouped according to the drainage technique received. Group A comprised 39 patients treated with conventional ultrasound-guided drainage from January 2010 to March 2019. Group B comprised 28 patients treated with CEUS-assisted drainage from August 2018 to January 2025. All patients received antituberculosis treatment.

Definitive surgery was subsequently performed in 30 patients in Group A and 23 patients in Group B. The remaining nine and five patients, respectively, did not undergo surgery because their condition improved after drainage or individualized assessment supported nonoperative management. All 67 patients were included in baseline and catheter-related analyses; surgery-specific outcomes were analyzed in the 53 patients who underwent surgery. The cohort underwent 72 drainage procedures: 41 procedures in 39 Group A patients and 31 procedures in 28 Group B patients.

This retrospective study was approved by the Ethics Committee of Hangzhou Red Cross Hospital (approval No. YS120-001) and was conducted in accordance with the Declaration of Helsinki. Written informed consent for ultrasound-guided catheter drainage was obtained from all patients before the procedure. The requirement for additional informed consent for this retrospective analysis was waived by the ethics committee because anonymized clinical data were used.

### Inclusion and exclusion criteria

Inclusion criteria were: (1) clinical, laboratory, and CT or MRI findings consistent with lumbar tuberculosis and PTA, with final microbiological confirmation by a positive Mycobacterium tuberculosis culture or Xpert result; (2) clear ultrasonographic visualization of an abscess with a transverse diameter of at least 3 cm; and (3) an indication for spinal tuberculosis surgery, including marked bone destruction, vertebral instability, spinal cord compression, or lower-limb dysfunction.

Exclusion criteria: (1) Patients with contraindications for lumbar tuberculosis surgery. (2) Patients with other organ diseases who could not tolerate anesthesia or surgery due to high risk. (3) Patients with active pulmonary tuberculosis whose lesions have not been controlled. (4) Patients with mental abnormalities who cannot cooperate. (5) Patients with coagulation abnormalities, severe skin ulcers, or sinus tract formation. (6) Patients whose imaging findings suggested hemangioma, lymphangioma, necrotic tumor, massive hematoma, or another non-tuberculous lesion rather than a paravertebral tuberculous abscess.

### Ultrasound-guided catheter drainage procedure

A Philips iU22 ultrasound system with 3.5–5 MHz, 5–12 MHz, and 3–9 MHz probes was used. Patients were examined in prone, lateral, and supine positions. Conventional ultrasonography assessed abscess extent and adjacent vessels and organs in both groups. Group A underwent skin preparation, local anesthesia, and ultrasound-guided percutaneous catheter drainage. In Group B, 2.4 mL of SonoVue (Bracco SpA, Milan, Italy) was injected through an antecubital vein, followed by a 5-mL saline flush. Contrast inflow and washout in the target cavity were observed for 2 min, and the CEUS recording guided target selection. Representative CEUS and corresponding B-mode images are shown in Figure 2. Catheter placement then followed the same preparation and ultrasound-guided technique used in Group A. Both groups received an 8F multifunctional drainage catheter (Bont, USA), which was fixed to the skin and connected to a drainage bag. Drained material was collected for smear microscopy, mycobacterial culture, and Xpert testing. Antituberculosis treatment continued after catheter placement. The same removal criteria were applied in both groups: drainage below 50 mL/day for 3 consecutive days, predominantly clear fluid, and no obvious residual pus on ultrasonography. Bilateral psoas abscesses were drained on both sides. Procedures were performed by members of the same interventional ultrasound team using a standardized technique, not by a single operator. Operator experience increased during the 15-year study period.

**Figure 1:**
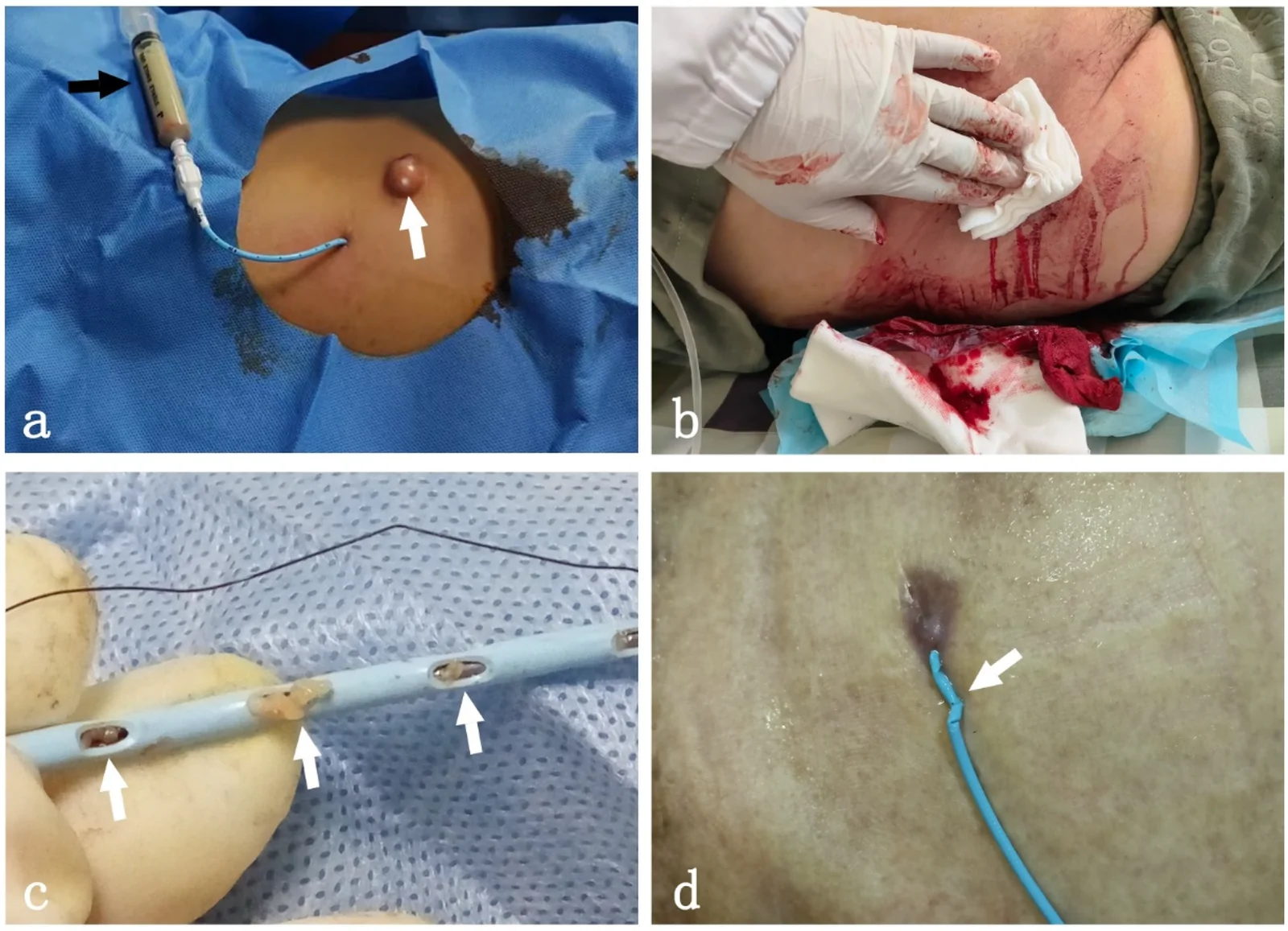
**(a)** 35-year-old man who underwent catheter drainage for a PTA at another hospital 3 years earlier, with the exact drainage duration unknown. One week before presentation, CT revealed a recurrent PTA, and he was referred to our hospital for further treatment. Ultrasonography demonstrated a PTA with a sinus tract (white arrow). Subsequently, ultrasound-guided catheter drainage was performed, yielding purulent fluid (black arrow). **(b)** A 39-year-old man with a PTA detected on MRI who underwent ultrasound-guided catheter drainage. One week after the procedure, the catheter was removed in preparation for surgical treatment; bleeding occurred after catheter removal and was successfully controlled with 1 h of manual compression. **(c)** A 60-year-old man with clinically confirmed PTA. After 1 month of catheter drainage, granulation tissue was observed within the catheter lumen at the time of catheter removal (white arrow). **(d)** A 69-year-old man with a PTA. More than 1 month after catheter placement, marked catheter kinking was observed, resulting in impaired drainage.

**Figure 2:**
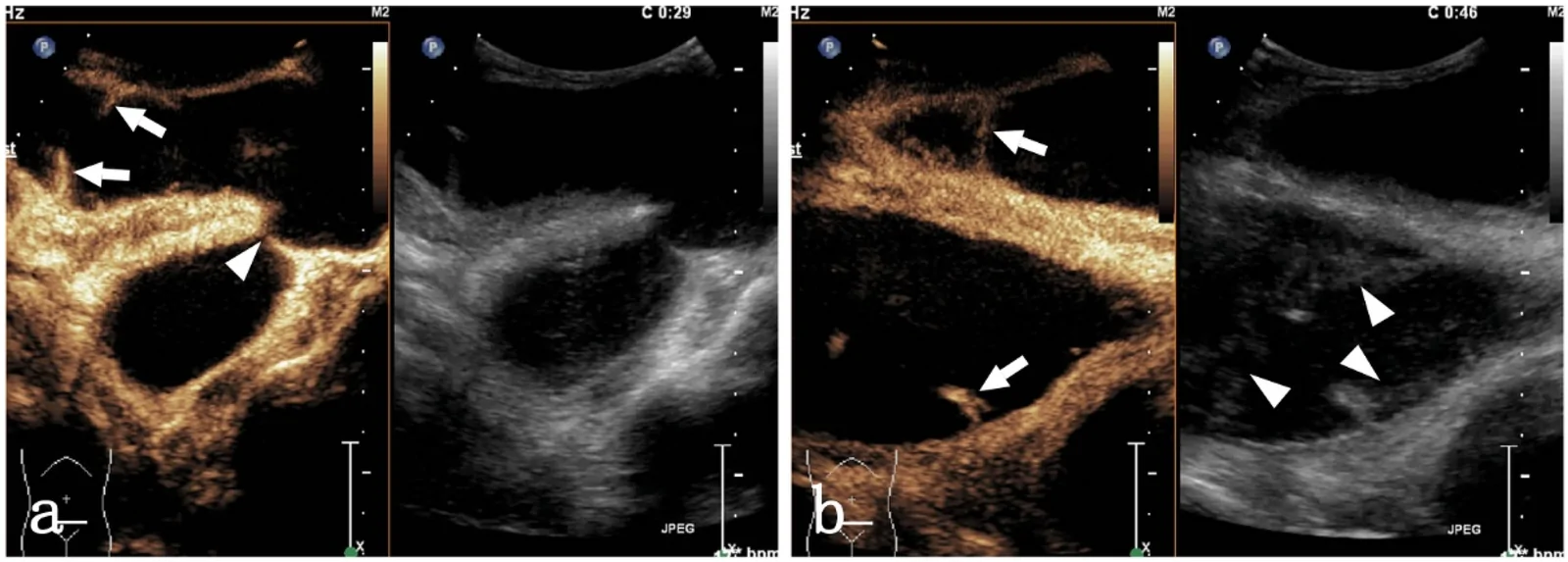
A 42-year-old woman with two left-sided PTAs. **(a)** CEUS image (left) and corresponding grayscale B-mode image (right) show the first abscess, with septal enhancement on CEUS (arrow) and thick pus and necrotic debris on B-mode imaging (arrowheads). **(b)** CEUS image (left) and corresponding B-mode image (right) show the second imaging plane, with internal septal enhancement (arrow) and poorly transmitting debris (arrowheads).

Complication assessment:

(1) Pain was assessed using a 0–10 visual analogue scale (VAS): 0 indicated no pain, 1–3 mild pain, 4–6 moderate pain, and 7–10 severe pain.
(2) Bleeding was assessed within 1 h after drainage. Bleeding comprised blood-stained drainage fluid, active bleeding at the catheter site after removal, or intra-abdominal bleeding identified by imaging or clinical findings.
(3) Difficulty in catheter removal was defined as failure to remove the catheter smoothly after releasing the fixation device and sutures.
(4) Cure was defined as resolution of tuberculosis-related symptoms, normalization or a marked decrease in ESR and CRP, and disappearance or reduction of the abscess cavity on ultrasonography without new destruction at the last recorded follow-up.
(5) Recurrence was defined as a new or enlarging abscess, sinus tract formation, or worsening neurological symptoms after initial improvement, confirmed by ultrasonography and requiring further treatment. Cure and recurrence were mutually exclusive. Complications were recorded separately and could coexist with either outcome. Outcomes were determined from the last available clinical, laboratory, and imaging record through April 2026.

### Statistical analysis

Data were analyzed using SPSS version 26.0. Continuous variables are presented as mean ± standard deviation. Normally distributed variables were compared using independent-samples t tests; non-normally distributed variables were compared using Mann–Whitney U tests. Categorical variables were compared using chi-square tests or two-sided Fisher's exact tests when expected cell counts were below 5. Table 4 used drainage procedures as the unit of analysis. Table 3 presents descriptive pre-treatment and 1-month values and between-group comparisons; no paired change analysis was performed. All tests were two-sided, and P < 0.05 was considered statistically significant.

## Results

### Baseline characteristics

The study included 67 patients. Baseline demographic and clinical characteristics are summarized in Table 1. Mycobacterium tuberculosis culture was positive in 12 patients, Xpert was positive in all 67 patients, and both tests were positive in 12 patients. Histopathological findings supported the diagnosis in all 67 patients.

**Table 1:** Baseline characteristics of the study population.

Characteristic	Category	Group A (39)	Group B (28)
Age		32.1 ± 6.1	31.0 ± 7.3
Gender	Male	24 (61.54%)	16 (57.14%)
	Female	15 (38.46%)	12 (42.86%)
Location of the occurrence	Bilateral	1 (2.56%)	3 (10.71%)
	Unilateral	38 (97.44%)	25 (89.29%)
Clinical signs and symptoms	Low fever and night sweats	16 (41.03%)	13 (46.43%)
	Numbness and pain in the lower limbs	4 (10.26%)	2 (7.14%)
	Progressive lower limb dysfunction	2 (5.13%)	1 (3.57%)

### Clinical and postoperative outcomes

Table 2 summarizes outcomes among the 53 patients who underwent definitive surgery, including 30 patients in Group A and 23 in Group B. Operative time, length of hospital stay, and intraoperative blood loss did not differ between groups. Cure and recurrence were assessed only in these 53 patients. Twenty-eight Group A patients and 22 Group B patients met the cure definition; recurrence occurred in two and one patients, respectively.

**Table 2:** Operative and postoperative outcomes among patients who underwent definitive surgery.

Group	Number of surgical cases	Blood loss (x¯ ± s, mL)	Surgical duration (x¯ ± s, min)	Hospitalization duration (x¯ ± s, days)	Clinical efficacy		
					Cure	Recurrent	Complication
Group A (*n* = 30)	30	483 ± 126	206 ± 58	21.5 ± 3.2	28	2	1
Group B (*n* = 23)	23	457 ± 97	183 ± 70	20.1 ± 2.6	22	1	0
Test value	0.269	0.849	1.307	1.709	0.395	0.095	0.781
*p* value	0.604	0.396	0.197	0.094	0.530	0.758	0.377

Analyses in this table were restricted to patients who underwent definitive surgery (Group A, *n* = 30; Group B, *n* = 23).

Table 3 presents ESR, CRP, and VAS values before treatment and at 1 month among the 53 patients who underwent definitive surgery. Mean values were lower at 1 month in both groups. Between-group differences were not significant at either assessment.

**Table 3:** Inflammatory markers and pain scores before treatment and at 1 month.

Group	Number of surgical cases	ESR (mm/h)		CRP (mg/L)		VAS (score)	
		Pretreatment	1 month after treatment	Pretreatment	1 month after treatment	Pretreatment	1 month after treatment
Group A (*n* = 30)	30	45.36 ± 10.35	26.23 ± 11.76	46.67 ± 28.36	19.03 ± 9.03	6.62 ± 0.59	1.95 ± 0.65
Group B (*n* = 23)	23	44.21 ± 11.39	27.50 ± 8.23	41.14 ± 24.81	18.50 ± 6.12	6.54 ± 0.74	2.18 ± 0.82
*t* value		0.428	−0.520	0.828	0.284	0.488	−1.283
*p* value		0.670	0.605	0.411	0.777	0.627	0.204

ESR, erythrocyte sedimentation rate; CRP, C-reactive protein; VAS, visual analog scale.

**Table 4:** Drainage-related complications. Values are numbers of drainage procedures (%), based on 41 procedures in Group A and 31 procedures in Group B. P values were calculated using two-sided Fisher's exact tests.

Complication	Group A (39 patients/41 procedures)	Group B (28 patients/31 procedures)	Statistical test	P value
Drain site pain	2 (4.88%)	1 (3.23%)	Fisher exact	1.000
Catheter occlusion	5 (12.20%)	3 (9.68%)	Fisher exact	1.000
Bleeding	5 (12.20%)	0 (0.00%)	Fisher exact	0.066
Catheter dislodgement	2 (4.88%)	1 (3.23%)	Fisher exact	1.000
Pericatheter purulent discharge	3 (7.32%)	1 (3.23%)	Fisher exact	0.629
Intra-abdominal infection	1 (2.44%)	0 (0.00%)	Fisher exact	1.000
Lumbar artery injury	1 (2.44%)	0 (0.00%)	Fisher exact	1.000
Difficulty in catheter removal	1 (2.44%)	1 (3.23%)	Fisher exact	1.000
Suture entrapment	1 (2.44%)	0 (0.00%)	Fisher exact	1.000

### Drainage-related complications

Group A included 39 patients and 41 drainage procedures. One patient underwent repeat drainage after accidental catheter removal, and one patient underwent bilateral drainage. Catheter dwell time was 7–21 days (mean, 11.0 +/- 2.4 days). Five bleeding events were recorded: four episodes of blood-stained drainage resolved within 1 h with observation, and one approximately 50-mL catheter-site bleed stopped after 1 h of local compression. Five procedures had catheter occlusion, two had drain-site pain, and one had difficulty in catheter removal. The recorded management was conservative except for surgical removal of the retained catheter.

Group B included 28 patients and 31 drainage procedures. Three patients underwent bilateral drainage. Catheter dwell time was 7–18 days (mean, 9.7 ± 3.7 days). One procedure had catheter occlusion, two had catheter deformation caused by positional compression, and one had mild drain-site pain that resolved after catheter repositioning. No bleeding event was recorded. Representative drainage-related complications are shown in Figure 1.

## Discussion

Ultrasound-guided catheter drainage reduces abscess burden before definitive treatment and avoids the trauma of open drainage (7, 8). Real-time imaging identifies the abscess, adjacent vessels, and nerve roots during catheter placement (9). It also avoids ionizing radiation, which is relevant when repeated procedures are required (10). These features support its use in selected patients with spinal tuberculosis and PTA.

Needle aspiration may be inadequate when pus is viscous or contains necrotic tissue and bone fragments (11, 12). In this cohort, thick material impeded aspiration in two patients, and two patients developed sinus tracts after repeated puncture. Catheter drainage provides continuous evacuation through multiple side holes and avoids repeated needle aspiration.

Catheter position affects drainage. Placement within a well-liquefied compartment promotes evacuation and reduces residual undrained pockets. Previous reports recommend entering through intact skin and positioning the catheter within a dependent drainable component (13, 14). In this cohort, neurological symptoms improved after abscess decompression.

A drainage catheter can also provide access for local irrigation. Local postoperative irrigation with antituberculosis drugs has been described in spinal tuberculosis (15). No patient in this cohort received local antituberculosis drug instillation.

Drainage-related complications included pain, catheter occlusion, bleeding, dislodgement, infection, sinus tract formation, and pericatheter discharge (16). Three patients reported mild drain-site pain. Repositioning the catheter or cushioning the fixation site relieved the symptoms.

Catheter occlusion can result from compression, viscous pus, necrotic debris, or granulation tissue (17). Eight occlusion or deformation events were recorded. Positional kinking resolved after repositioning, and obstruction by viscous pus resolved after saline irrigation. Granulation tissue was identified in two catheters retained for more than 1 month. Negative-pressure aspiration or irrigation can restore drainage when intraluminal obstruction occurs (18). Related studies have described the clinical characteristics of spinal tuberculosis and CT-guided catheter drainage (19, 20), while CEUS has also been used to guide biopsy of thoracic and peripheral pulmonary lesions (21, 22).

Both groups received the same 8F catheter and followed the same removal criteria. Mean catheter dwell time was 11.0 days in Group A and 9.7 days in Group B. CEUS delineated liquefied or necrotic components, vascularized tissue, and internal septa before placement. This information supported selection of a larger, more drainable target compartment and may explain the shorter dwell time in Group B. Length of hospital stay did not differ between groups. Patient discomfort, nursing workload, and catheter-related costs were not measured.

CEUS depicts vessels in the abscess wall and along the planned catheter path. In this cohort, bleeding occurred after 5 of 41 conventional procedures and after 0 of 31 CEUS-assisted procedures (Fisher's exact *P* = 0.066). Four events resolved within 1 h with observation, and one approximately 50-mL bleed stopped after local compression. The lower recorded event rate was an exploratory association based on five events.

Catheter dislodgement, intra-abdominal infection, sinus tract formation, and pericatheter discharge were uncommon. Standardized fixation, path planning, catheter care, and aseptic technique were used in both groups.

This study has four main limitations. First, CEUS use was linked to a later treatment period, so temporal changes in operator experience, equipment, catheter care, and clinical management could not be separated from the imaging strategy. Second, historical records did not consistently contain abscess volume, loculation, vertebral involvement, bone destruction, comorbidities, or complete hematological and coagulation data. Third, follow-up intervals were not uniform, and surgery-specific outcomes were available only for the 53 patients who underwent definitive surgery. Fourth, the sample was small, no *a priori* sample size calculation was performed, and only five bleeding events occurred. These features limit adjusted analysis and causal interpretation.

## Conclusion

In this retrospective historical cohort, CEUS-assisted drainage was associated with fewer recorded bleeding events and provided additional anatomical information for catheter planning.

## Data Availability

The raw data supporting the conclusions of this article will be made available by the authors, without undue reservation.
